# Vestibular Hair Cells Require CAMSAP3, a Microtubule Minus-End Regulator, for Formation of Normal Kinocilia

**DOI:** 10.3389/fncel.2022.876805

**Published:** 2022-06-17

**Authors:** Josephine O’Donnell, Jing Zheng

**Affiliations:** ^1^Department of Otolaryngology, Feinberg School of Medicine, Northwestern University, Chicago, IL, United States; ^2^Knowles Hearing Center, Northwestern University, Evanston, IL, United States

**Keywords:** CAMSAP3, kinocilia, “9+2” configuration, gait, vestibular function, kidney dysfunction

## Abstract

Kinocilia are exceptionally long primary sensory cilia located on vestibular hair cells, which are essential for transmitting key signals that contribute to mammalian balance and overall vestibular system function. Kinocilia have a “9+2” microtubule (MT) configuration with nine doublet MTs surrounding two central singlet MTs. This is uncommon as most mammalian primary sensory cilia have a “9+0” configuration, in which the central MT pair is absent. It has yet to be determined what the function of the central MT pair is in kinocilia. Calmodulin-regulated spectrin-associated protein 3 (CAMSAP3) regulates the minus end of MTs and is essential for forming the central MT pair in motile cilia, which have the “9+2” configuration. To explore the role of the central MT pair in kinocilia, we created a conditional knockout model (cKO), *Camsap3-*cKO, which intended to eliminate CAMSAP3 in limited organs including the inner ear, olfactory bulb, and kidneys. Immunofluorescent staining of vestibular organs demonstrated that CAMSAP3 proteins were significantly reduced in *Camsap3-*cKO mice and that aged *Camsap3-*cKO mice had significantly shorter kinocilia than their wildtype littermates. Transmission electron microscopy showed that aged *Camsap3-*cKO mice were in fact missing that the central MT pair in kinocilia more often than their wildtype counterparts. In the examination of behavior, wildtype and *Camsap3-*cKO mice performed equally well on a swim assessment, right-reflex test, and evaluation of balance on a rotarod. However, *Camsap3-*cKO mice showed slightly altered gaits including reduced maximal rate of change of paw area and a smaller paw area in contact with the surface. Although *Camsap3-*cKO mice had no differences in olfaction from their wildtype counterparts, *Camsap3-*cKO mice did have kidney dysfunction that deteriorated their health. Thus, CAMSAP3 is important for establishing and/or maintaining the normal structure of kinocilia and kidney function but is not essential for normal olfaction. Our data supports our hypothesis that CAMSAP3 is critical for construction of the central MT pair in kinocilia, and that the central MT pair may be important for building long and stable axonemes in these kinocilia. Whether shorter kinocilia might lead to abnormal vestibular function and altered gaits in older *Camsap3-*cKO mice requires further investigation.

## Introduction

Cilia, found on cell surfaces, are microtubule (MT)-based organelles that play essential roles for cell development, proliferation, differentiation, migration, signal transduction, etc. The structure, length, and function of cilia must be tightly regulated because their dysfunction is associated with numerous diseases collectively called ciliopathy disorders (Lee and Gleeson, 2011; Falk et al., 2015; Reiter and Leroux, 2017; Andreu-Cervera et al., 2021; Lee and Ostrowski, 2021). Based on their mobility, cilia are divided into two types: motile cilia and non-motile cilia like primary cilia. Motile cilia beat rhythmically to transport fluids across epithelia, while non-motile, as known as primary cilia, serve as sensory organelles gathering information about their environment. The majority of motile cilia in mammals are composed of MTs in the “9+2” configuration, i.e., nine doublet MTs surrounding two central singlet MTs. In contrast, most of the primary sensory cilia in mammals do not have central MT pairs, and instead, their axonemes have a “9+0” configuration.

Vestibular kinocilia, located on hair cells of the vestibular organ, are primary sensory cilia present throughout the murine lifespan. Compared to other primary sensory cilia, vestibular kinocilia are exceptionally long, some being 3–4 times longer than adjacent microvilli called stereocilia (Xue and Peterson, 2006). The actin-based stereocilia and MT-based kinocilia on vestibular hair cells are connected by lateral links and tip links (Figure 3B). Because of their length, the tips of vestibular kinocilia reach into the overlying otoconial layer. This structural arrangement allows vestibular kinocilia to transmit positions and movements of the head by change the otoconial membrane mass to the adjacent stereocilia, which generate electrical signals in sensory hair cells and ultimately controls mammalian balance and awareness of spatial orientation. As such, the mechanical properties and length of vestibular kinocilia play important roles in determining the operating range of the vestibular system (Spoon and Grant, 2011, 2013; Nam, 2018). Unlike most of primary sensory cilia, vestibular kinocilia have the “9+2” configuration, a common configuration found in motile cilia (Wersall et al., 1965; Rosenhall and Engstrom, 1974; Choksi et al., 2014). Unlike the outer 9 MT doublets, the central MT pair is not continuous with MT triplets in the basal body (Lechtreck et al., 2013). In motile cilia, the central MTs and their MT-associated proteins are required for the synchronized motion needed to remove debris (Loreng and Smith, 2017). The function of central MTs in primary cilia remains unknown. Primary cilia on olfactory sensory neurons and vestibular hair cells have the longest axoneme in mammals. Intriguingly, both cilia have the “9+2” configuration, which leads us to suspect that the central MT pair may be essential for the formation of the long axoneme.

**FIGURE 1:**
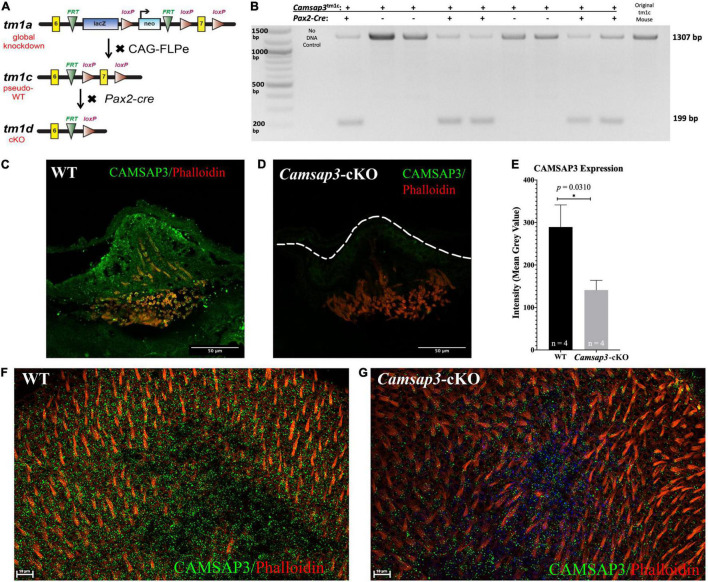
Creation and validation of the *Camsap3-*cKO mouse. **(A)** A schematic diagram of the *Camsap3* knockout first targeting strategy and conversion of tm1a (KO first) to tm1c (floxed), and tm1d (cKO) alleles, respectively. Tg*Pax2*^Cre^**^/+^; *Camsap3*^tm 1^*^c/tm^*^1^*^c^* mice, known as *Camsap3*-cKO, are a cKO model for the vestibular organ. **(B)** The exon 7 of *Camsap3* execution was validated by PCR genotyping. The inner ear genomic DNA of offspring derived from crossing Tg*Pax2*^Cre^** and *Camsap3*^tm 1^*^c/tm^*^1^*^c^* were isolated and used for PCR (*n* = 9 from two separate litters). No DNA and floxed allele *Camsap3*^tm 1^*^c/tm^*^1^*^c^* were used as the negative and positive controls. The bands were expected to be 1307 bp for WT (Tg*Pax2^Cre+/+^*; *Camsap3*^tm 1*c*/*tm*1*c*^) and 199 bp for *Camsap3*-cKO mice. **(C–G)** CAMSAP3 protein expression in the vestibular system as examined by immunofluorescence. **(C,D)** Representative immunofluorescent images of a crista from WT **(C)** and *Camsap3*-cKO **(D)** mouse at P75 were shown. The dashed line outlines the edge of the crista of a *Camsap3*-cKO mouse. Scale Bars = 50 μm. **(E)** Vestibular cells from *Camsap3*-cKO mice had significantly less CAMSAP3 staining than their WT counterparts. The bars represent mean ± SD. *Statistically significant difference (*p* = 0.03). WT and *Camsap3*-cKO mice littermate (P75) replicates (n) were as indicated. **(F,G)** Representative confocal maximum projection of z-stack images taken from whole-mounts utricle samples of a WT **(F)** and a *Camsap3*-cKO mouse **(G)**. More CAMSAP3 staining dots were found on the apical surface of WT utricle compared to those on *Camsap3*-cKO mouse. Scale Bars = 10 μm. Antibodies: anti-Camsap3 (Green), phalloidin (Red).

**FIGURE 2:**
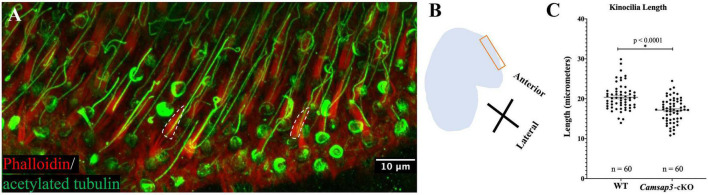
Kinocilia on utricles hair cells from *Camsap3*-cKO are shorter than their WT littermates. **(A)** A representative 3D-reconstruction from z-stacks images of bundles of stereocilia (red) and kinocilia (green) located on the apical surface of hair cells from an utricle of a WT mouse (P386). Antibodies: anti-acetylated tubulin (Green), phalloidin (Red). The dashed line outlines the edge of actin-based stereocilia bundles. Scale Bar = 10 μm. **(B)** A schematic diagram showing the region over which Z-stack images of utricle hair cells were collected. **(C)** Quantification of kinocilia length collected from male WT (*n* = 3) and male *Camsap3*-cKO littermates (*n* = 3) with ages ranging from 10- to 13-month. Each dot represented one kinocilium. Bars represent mean ± SD. Kinocilia on utricles hair cells from *Camsap3*-cKO were statistically significant shorter than WT (*p* < 0.0001). *Statistically significant difference.

**FIGURE 3:**
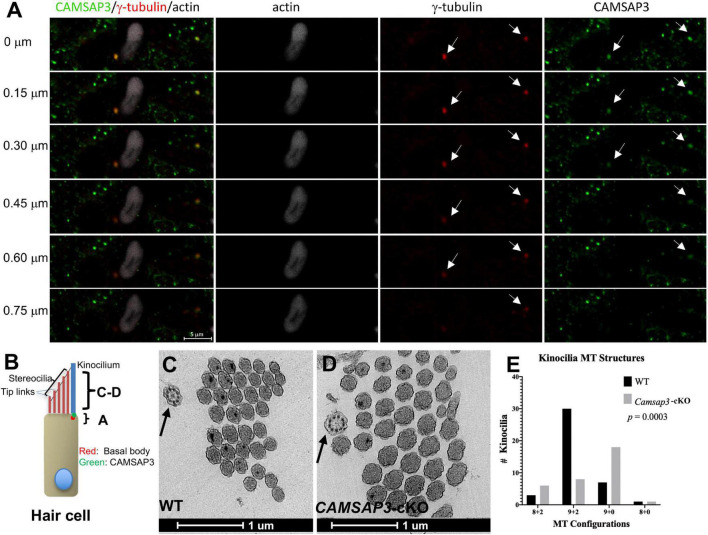
CAMSAP3 contributes to the formation of central MT pairs in axonemes of kinocilia on utricle hair cells. **(A)** The overlaps between CAMSAP3 (green) and basal bodies (red) were displayed by a group of consecutive Z-stack kinocilia images taken from a whole mount utricle sample of WT (P42). Z-stack images were captured using the optical section (0.5 μm) starting from the apical surface of utricle hair cells toward its nucleus. Antibodies: anti-CAMSAP3 (Green), anti-γ-tubulin (red), phalloidin (white). Scale Bars = 5 μm. Two basal bodies of kinocilia were indicated by arrows. **(B)** A schematic diagram illustrates the region over which Z-stack images of kinocilia immunostaining A, and TEM images C-D were collected. **(C,D)** Representative TEM images show transverse sections of kinocilia on utricle hair cells from 8-month-old WT with a “9+2” configuration (black arrow) **(C)**, and a “9+0” configuration (black arrow) for their *Camsap3*-cKO littermates **(D)**. Scale Bars = 1 μm. **(E)** The distribution of MT arrangements for both WT and *Camsap3*-cKO mice was compared. Distribution of the MT configurations between WT and KO was significantly different as analyzed by a Kolmogorov–Smirnov test (*p* = 0.0003).

Calmodulin-regulated spectrin-associated protein 3 (CAMSAP3), also called Marshalin (Zheng et al., 2013), is a MT minus-end regulator. Because CAMSAP3 can interact with other proteins through its protein-protein interaction domains, it has been reported to play several regulatory roles through interactions with multiple proteins (Meng et al., 2008; Goodwin and Vale, 2010; Toya et al., 2016; Gibieža et al., 2021). For example, CDH23 is an adhesive protein that plays crucial roles for hearing and balance. The C isoform of CDH23 can directly bind to CAMSAP3 and modifies CAMSAP3-associated MT networks (Takahashi et al., 2016). In addition, CAMSAP3 is involved in the formation of MTs from non-centrosomal MT organizing centers (MTOCs). CAMSAP3-coated MTs are stable and can be used to seed tubulin polymerization at non-centrosomal sites (Jiang et al., 2014, 2018; Zenker et al., 2017). We recently discovered that CAMSAP3 is necessary to form the central MT pairs in motile cilia (Robinson et al., 2020). In the *Camsap3*^tm 1^*^a/tm^*^1^*^a^* mice, the global knockdown (KD) of CAMSAP3 expression is associated with impaired ciliary motion, leading to phenotypes of Primary Ciliary Dyskinesia (PCD), which includes hydrocephalus, subfertility, and impaired mucociliary clearance. Dysfunctional mucociliary clearance leads to hyposmia, anosmia, rhinosinusitis, and otitis media. We occasionally noticed *Camsap3*^tm 1^*^a/tm^*^1^*^a^* mice (*Camsap3-*KD for short) with a head tilt to one side. Therefore, we suspect that CAMSAP3 is also needed to construct the central MT pair in kinocilia on vestibular hair cells and predict that the central MT pair in vestibular kinocilia is essential to establish the long and stable cilia required for vestibular function.

CAMSAP3 is expressed ubiquitously in mice. The global KD and global knockout (KO) CAMSAP3 in mice could potentially affect all cells that express CAMSAP3. Indeed, removal of *Camsap3* gene from a mouse genome leads to global *Camsap3*-KO mice dying prematurely (Muroyama et al., 2018). Even the *Camsap3-*KD mice, which reduce CAMSAP3 expression through RNA processing (Testa et al., 2004; White et al., 2013) without deleting any exons of *Camsap3* gene, showed some degree of embryonic lethality, sub-infertility, hydrocephalus, anosmia or hyposmia, sinusitis, respiratory distress, otitis media, and hearing loss (Perez-Garcia et al., 2018; Ingham et al., 2019; Robinson et al., 2020). To investigate the function of CAMSAP3 in a reliable living mouse model, we created a conditional knockout (cKO) mouse model known as *Camsap3*^tm 1^*^d/tm^*^1^*^d^* (*Camsap3-*cKO for short) for tissue specific cKO by using Cre-loxP system.

*Camsap3-*cKO would allow us to examine CAMSAP3’s role in vestibular hair cells without influence from other health issues. Tg*Pax2-Cre* mouse line is widely used to establish cKO mouse models for inner ear (Pan et al., 2011; Cox et al., 2012; Wiwatpanit et al., 2018). In this transgenic mouse line, *IRES-Cre* was inserted into *Pax2* (paired box gene 2), a transcription factor that is crucial in the developmental and proliferation of multiple cells and organs. *Cre* expression was detected at E8.5 in the otic placode in the *Pax2*-*Cre* mouse line (Ohyama and Groves, 2004), which is earlier than when hair cell differentiation typically occurs (around E13.5) (Mbiene et al., 1984). Thus, we created *Camsap3-*cKO by crossing Tg*Pax2-Cre* mice with *Camsap3*^tm 1^*^c/tm^*1*^c^* (fl/fl), aiming to eliminate CAMSAP3 expression in *Cre*-expressed tissues, which include most of the cells in the inner ear, midbrain, cerebellum, the olfactory bulb, and kidneys (Ohyama and Groves, 2004). If CAMSAP3 is important for establishing normal vestibular kinocilia as we predict, we expect *Camsap3*-cKO mice to show behaviors associated with vestibular dysfunction. Different assessments, including Rotarod balance tests, DigiGait analyses, along with anatomical examination, were used to characterize the vestibular functions of *Camsap3*-cKO mice. We also evaluated olfactory function and monitored kidney health in order to determine whether removing CAMSAP3 causes detrimental effects outside of our expected outcomes in the vestibular system. Our data suggests that CAMSAP3 has an important role in establishing or maintaining normal vestibular kinocilia and kidney function, but is not essential for the task of olfactory bulbs.

## Materials and Methods

### Creation of *Camsap3*-cKO Mice

All experiments utilizing animals were approved by the Institutional Animal Care and Use Committees of Northwestern University and in accordance with the National Institutes of Health guidelines. Animals were housed in Northwestern’s Center for Comparative Medicine facilities.

The *Camsap3*^tm 1^*^a (EUCOMM)^ ^Wtsi^* (referred to as *Camsap3-*KD) mouse model was obtained from the Wellcome Trust Sanger Institute. The original *Camsap3-*KD line on the C57B6N background was re-derived on FVB murine backgrounds to increase their viability (Robinson et al., 2020). As shown in Figure 1A, a *Camsap3-*KD mouse was created using the “knockout first, conditional-ready” strategy (Skarnes et al., 2011). A targeted trap allele was inserted into the intron between the exon 6 and 7 of the *Camsap3* gene to knock out *Camsap3* expression through RNA processing (Testa et al., 2004; White et al., 2013) without deleting any exons. To create a cKO mouse model, *Camsap3-*KD mice were first crossed with B6-Tg(CAG-FLPe)36 (Kanki et al., 2006) to delete the *neo* and *LacZ* cassettes flanked by FRT sites and create *Camsap3*^tm 1^*^c^* (floxed allele), a strain of pseudo-wildtype (WT) mice. This WT *Camsap3^tm 1^*^c^** floxed line was further crossed with the Tg*Pax2*^Cre^** mouse line, Tg(Pax2-*Cre*)1Akg/Mmnc (MMRRC stock number 10569) (Ohyama and Groves, 2004) to create a conditional knockout mouse model: Tg*Pax2*^Cre^**^/+^; *Camsap3*^tm 1^*^c/tm^*^1^*^c^* and their WT littermates: Tg*Pax2*^Cre+/+^*;Camsap3*^tm 1^*^c/tm^*^1^*^c^*. We call this mouse model *Camsap3*^tm 1^*^d/tm^*^1^*^d^* (null allele) *Camsap3-*cKO for short. All animals were maintained by heterotypic breeding, and the genotypes were determined by detecting the inserted cassettes in mouse tail samples performed by Transnetyx (Cordova, TN, United States).

Since *Cre* expression was limited in a few tissues including the inner ear, olfactory bulb, and kidneys of Tg*Pax2^Cre^* mice, the exon 7 of *camsap3* was expected to be fully removed from these tissues of *Camsap3-*cKO mice. To verify the exon 7 exclusion, genomic DNA was purified from the inner ear of WT and *Camsap3*-cKO mice using the QIAamp DNA FFPE Tissue Kit (Qiagen). PCR was used to verify the exon 7 exclusion using the PCR Master Mix (Thermo Fisher Scientific, A44647100) with forward primer L1L2-BactP-MD-F (GCTGGCGCCG GAACC) and reverse primer C3Floxed5-R (TTGGCCTGGGGAACATGAC). Conditions for the thermal cycler were: step 1–95°C, 2 min; step 2–95°C, 30 s; step 3–55°C, 30 s, step 4–72°C, 90 s; repeat steps 2–4, 35 × ; step 5–72°C, 10 min. Expected sizes were 1,307 base pairs (bp) for WT, and 199 bp for *Camsap3*-cKO mice. Both male and female mice were used in all experiments.

### Evaluation of Vestibular Function

#### Swimming Test

WT mice and their *Camsap3*-cKO littermates were placed in a 5-gallon bucket filled with room temperature water and were required to swim for 3 min. Behavior was observed, and any abnormal behavior was recorded. The swim test protocol was a modified version of a protocol described previously (Hardisty-Hughes et al., 2010).

#### Right Reflex Test

We modified a protocol described previously (Pau et al., 2004) to assess right reflexes. WT mice and their *Camsap3*-cKO littermates were placed in a clear plastic box (1′′× 2′′× 5′′). The box was quickly flipped upside down and the time it took each mouse to flip themselves back upright was recorded.

#### Rotarod Test

The rotarod test was created by Dunham and Miya (1957) to assess neuromuscular function in rodents and is commonly used to assess balance (Jones et al., 2018). The Rotarod apparatus (TSE Systems, Chesterfield, MO, United States) was used to assess balance in *Camsap3*-cKO and their WT littermates. Following the Jones et al. (2018) protocol, mice were placed on a rotating beam and their time to fall (TTF) was recorded. Mice were tested 3 at a time with their cage mates. The rotarod was set to start rotating at a speed of 5 rpm and accelerate to a top speed of 44 rpm over the course of 300 s (the maximum duration of the trial). The mice were given 10-min trial intervals and tested again. Ten *Camsap3*-cKO mice and 10 WT littermates completed eight trials, with the first three trials functioning as an acclimation and familiarization period. The final five trials were recorded for analysis.

#### Gait Analysis

The DigiGait™ Imaging System apparatus (Mouse Specifics Inc., Framingham, MA, United States) consists of a motorized transparent treadmill belt sitting above a digital camera which records the ventral view (Supplementary Movie 1). WT mice and their *Camsap3*-cKO littermates were placed on the treadmill one at a time and were required to run. The mice were then evaluated at treadmill speeds of 10, 17, and 24 cm/s. The DigiGait assay was run by Northwestern Behavioral Phenotyping Core. The DigiGait Imaging System software allowed us to examine 52 different aspects of each mouse’s gait (Rostosky and Milosevic, 2018). The analysis software can detect slight differences in angles, lengths, and speeds of the paws and strides of the mice. The key behaviors we were interested in, shown in Supplementary Figures 1, 2, included: swing to stance ratio, paw angle, gait symmetry, stance width, step angle, breaking duration, stride length, the ataxia coefficient, paw area, and the maximum change in paw area (maximum dA/dt).

### Immunofluorescence and Microscopy

The inner ears, olfactory bulbs, and olfactory mucosa in nasal cavities were collected from WT mice and their *Camsap3*-cKO littermates for immunofluorescence as previously described (Sekerka et al., 2011; Robinson et al., 2020). Briefly, samples were immersion fixed in 4% formaldehyde in phosphate buffered solution (PBS). Some samples underwent whole mount preparation, in which the utricles and cristae were further dissected out and used for immunostaining. Other samples were cryosectioned before staining. These samples were first decalcified in 0.125M EDTA for 2 days. Decalcified samples were then placed in a series of sucrose solutions in 1X PBS (10–30%) then two changes of Tissue-Tek Optimal Cutting Temperature (OCT) Embedding Medium (Sakura Finetek, 4583), and embedded in fresh OCT. The inner ear samples were then sectioned into 12 micrometer thick slices, and post-fixed in 2% formaldehyde for 10 min and blocked at room temperature for 1 h in blocking solution: 5% goat serum, 2% Triton X-100 in Tris-buffered saline (TBS). The following primary antibodies were used for immunostaining: anti-Camsap3 (Robinson et al., 2020) at 1:2500, anti-acetylated tubulin at 1:500 (Thermo Fisher Scientific 32-2700, AB_2533073). Samples were incubated with primary antibodies at 4°C overnight. The next day samples were then washed in PBS and incubated with appropriate fluorophore-conjugated secondary antibodies, including goat anti-rabbit Alexa 488 at 1:500 (Thermo Fisher Scientific 32-2700, AB_143165), goat anti-mouse Alexa Fluor 488 (RRID: AB_2556548), goat anti-mouse IgG2b Alexa 647 at 1:500 (Thermo Fisher Scientific AB_143165), Hoechst at 1:1000 (Thermo Fisher Scientific, H3570), and Phalloidin-Alexa 568 at 1:2000 (Thermo Fisher Scientific 21838, AB_2532159), or Phalloidin-Alexa 647 at 1:400 (Thermo Fisher Scientific A22287) for 2.5 h at room temperature. The stained samples were subsequently mounted in Fluoromount Aqueous Mounting Medium (Sigma, F4680). When mounting the whole mount utricle samples, a 0.12 mm deep Secure-Seal spacer (Thermo Fisher Scientific, S24735) was placed between the slide and the cover glass in order to avoid destruction of the vestibular kinocilia on hair cells. Immunostained samples were imaged using a Nikon C2 or A1R+ confocal microscope using objects of a plan Apo VC 20×/0.75 DIC N2 (Nikon) and a plan Apo 60×/1.4 oil (Nikon). All images were taken as 12-bit, which allows for 0–4,095 range. For vestibular kinocilia length measurement, Z-stacks images were captured from the apical surface of utricle hair cells to the tip of vestibular kinocilia (from the position “A” extended to “C-D” shown in Figure 3B) using the optical section (0.5 μm). These Z-stack images were then reconstructed into 3D images, which were analyzed using Imaris 8 (Bitplane) and Nikon NIS Element software. Mean gray value, the sum of the gray values of all the pixels in the selection divided by the number of pixels, was measured using FIJI.

### Transmission Electron Microscopy

Utricles from WT mice and their *Camsap3*-cKO littermates were dissected out and immersion fixed in 0.1 M sodium cacodylate buffer pH 7.3, containing 2% paraformaldehyde and 2.5% glutaraldehyde for at least 24 h and post-fixed with 2% osmium tetroxide followed by 3% uranyl acetate. Tissues were then dehydrated in ascending grades of ethanol, transitioned with propylene oxide and embedded in resin mixture from the Embed 812 kit, cured in a 60°C oven. Samples were sectioned on a UCT ultramicrotome (Leica Microsystems). To identify regions of interest 1 μm thick sections were collected, stained with Toluidine Blue O and examined by light microscopy. 70 nm tissue sections were collected on 200 mesh copper grids and stained with uranyl acetate and Reynolds lead citrate, and examined using a FEI Tecnai G2 Spirit Transmission Electron Microscope. The number of MTs in kinocilium was counted and sorted by structure: “9+2,” “9+0,” “8+2,” or “8+0.” The first number indicates the numbers of MT doublets outlying, and the second number implies the amount of MTs in the center.

### Evaluation of Olfaction

The Buried Food Test was used to evaluate olfaction in mice (Yang and Crawley, 2009; Robinson et al., 2020). WT mice and their *Camsap3*-cKO littermates were food restricted but provided water *ad libitum* for 24 h prior to testing. Mice were given a piece of a Nutter Butter cookie during this day to familiarize the mice with its scent. Testing was performed during daylight, starting at 9 AM. The base of a Sterilite translucent storage box (cage) [47 cm (L) × 37.8 cm (W) × 28.3 cm (H)] was filled with bedding to a depth of 3 cm. Each mouse was placed in an acclimation bin which was identical to the testing bin in every way, with the exception of no food being present, and were given 5 min to adjust to the enclosure. Mice were then transferred individually to the testing cage, which had ∼ 0.5 g of Nutter Butter cookie hidden in the center of the cage under the bedding. As soon as the mouse entered the testing bin a stopwatch was started and the latency to find (LTF) the cookie was recorded. Following the experiment, mice were returned to their home cages and given their full allotment of food.

## Results

### *Camsap3*-cKO Is a *Camsap3* Conditional Knockout Model for the Vestibular Organ

As previously reported, the global KD mice, *Camsap3-*KD, were born with smaller bodies along with sub-infertility, hydrocephalus, anosmia or hyposmia, sinusitis, respiratory distress, otitis media, and hearing loss (Perez-Garcia et al., 2018; Ingham et al., 2019; Robinson et al., 2020). In contrast to *Camsap3-*KD*, Camsap3*-cKO had normal fertility and a normal appearance comparable to their WT littermates regardless of their sex prior to 4 months of age, The normal appearance of *Camsap3*-cKO was not surprising as *Camsap3* was expected to be eliminated only in the inner ear, midbrain, cerebellum, olfactory bulb, and the kidneys (Ohyama and Groves, 2004). To test whether the exon 7 of *Camsap3* was indeed removed from the genome of affected cells, we performed PCR genotyping using genomic DNA isolated from the inner ear, where *Cre* was expressed to remove the floxed exon 7 of *Camsap3*. As shown in Figure 1B, in all *Camsap3*-cKO mice (*Pax2-Cre* positive), the expected short fragments (199 bps) were detected, but not in mice without the *Pax2*-*Cre* allele (*Pax2-Cre* negative) or original homozygous *Camsap3*^tm 1^*^c^* mice. We noted that the long PCR band, 1307 bp, was also detected in *Camsap3*-cKO mice with low intensity. As we did not perform cardiac perfusion before extracting genomic DNA from the inner ear, we suspected that cells from the circular bloodstream in the inner ear could contribute to the long PCR bands as the genomic DNA from these cells were not affected by *Pax2*-*Cre* expression. It is also possible that the exon 7 of *Camsap3* was not completely removed from all cells in the inner ear. To verify whether *Camsap3* was expressed in the vestibular hair cells, we performed immunofluorescence using vestibular organs collected from *Camsap3*-cKO and their WT littermates, with the ages of postnatally day (P) 23 to 260. In WT, CAMSAP3 expression was found in the vestibular organ including the hair cells in cristae (Figure 1C), utricle (Figure 1F), and saccule (data not shown). CAMSAP3 expression in *Camsap3*-cKO was absent or reduced in vestibular cells from *Camsap3*-cKO mice as shown in Figures 1D,G. CAMSAP3 staining was expressed in both hair cells and their surrounding supporting cells in vestibular organs from WT samples. CAMSAP3 signals were more concentrated in the apical cortical area of the WT vestibular organ (Figure 1C). Such distribution patterns are unsurprising as CAMSAP3 is needed at apical cortical area of epithelial cells to establish the intercellular connection and orient the apical-to-basal polarity of microtubule arrays (Meng et al., 2008; Toya et al., 2016). We further quantified CAMSAP3 staining intensities in the apical cortical area that included both vestibular hair cells and supporting cells. The mean gray value (intensity) of CAMSAP3 staining in these areas were measured using FIJI software. As shown in Figure 1E, CAMSAP3 expression was significantly decreased in the hair cell area and supporting cells from *Camsap3*-cKO mice, with CAMSAP3 expression in *Camsap3*-cKO mice equaling 48.74% of expression in WT mice at P75 (*p* = 0.031, unpaired *t*-test, *n* = 4). In sum, we created a cKO model that significantly reduced CAMSAP3 proteins in the vestibular system.

### *Camsap3*-cKO Mice Have Abnormal Kinocilia on Vestibular Hair Cells

To investigate CAMSAP3’s role in establishing kinocilia on the vestibular hair cells, utricles collected from *Camsap3*-cKO mice (3 male) and their WT littermates (3 male) around 1-year old (P296-P384) were examined using immunofluorescent staining of anti-acetylated tubulin and phalloidin, which labeled vestibular kinocilia (green) and stereocilia (red, dash lines), respectively (Figure 2A). We measured the length of kinocilia on vestibular hair cells of utricles from *Camsap3*-cKO mice and their WT littermates at the most anterior portion for consistency (Figure 2B). From each mouse sample, the length of kinocilium was measured from base to tip in micrometers. Great care was taken to exclude vestibular kinocilia whose full length was not captured in the confines of the reconstructed 3D image. The average length of the vestibular kinocilia, in mean ± SD, were 20.39 μm ± 3.21 for WT and 17.16 μm ± 3.03 for *Camsap3*-cKO (Figure 2C). An unpaired *t*-test revealed a significant reduction in vestibular kinocilia length in *Camsap3*-cKO mice when compared with their WT littermates (*p* < 0.0001).

Our previous work demonstrated that CAMSAP3 was needed for *de novo* formation of the central MT pair in a “9+2” configuration observed in the axons of motile cilia (Robinson et al., 2020). To test whether CAMSAP3 was also needed for formation of the central MT pair in the kinocilia on vestibular hair cells, we first examined the connection between CAMSAP3 and basal bodies by immunofluorescence. The basal bodies that extend to 9 peripheral MT doublets were labeled by anti-γ-tubulin, and phalloidin was used to label the actin-based stereocilia that were adjacent to the kinocilium. As shown in Figure 3A, a group of Z-stack images taken from the apical surface of utricle hair cells (the position “A” shown in Figure 3B) showed two vestibular kinocilia labeled by γ-tubulin (arrows). A stereocilia bundle was next to one of kinocilia, indicating these cells were hair cells from an utricle. Similar to other epithelial cells, CAMSAP3 punctuates (green) were found on the apical surface of hair cells. Two CAMSAP3-stained green dots (arrows) were colocalized with two basal bodies (red dots). As the focus pointing moved step by step (0.5 μm/per section) from the apical surface toward the nucleus, green CAMSAP3 dots (arrows in Figure 3A) in both vestibular kinocilia disappeared before the red basal bodies punctuates, suggesting that CAMSAP3 was at the base of vestibular kinocilia just above the basal bodes, as illustrated in Figure 3B. Such a close connection between CAMSAP3 and basal bodies was of particular interest because CAMSAP3 is not a centrosome- or basal bodies-bound protein. In fact, CAMSAP3 was not observed in the basal bodies of primary cilia with “9+0” MT configuration (Robinson et al., 2020). More importantly, the close association between γ-tubulin and CAMSAP3 was comparable to that observed in motile cilia with “9+2” MT configuration (Robinson et al., 2020; Saito et al., 2021), suggesting that CAMSAP3 was also involved in *de novo* formation of the central MT pair in the kinocilia on the vestibular hair cells.

To further verify the role of CAMSAP3 in formation of the central MT pair in vestibular kinocilia, the MT structure differences between WT and *Camsap3*-cKO vestibular kinocilia were then evaluated using TEM (Figures 3C–E). We examined 42 axoneme structures (the position “C-D” shown in Figure 3B) from 8-month-old WT mice and 33 axonemes from their *Camsap3*-cKO littermates. The “9+2” configuration here includes 9 MT doublets peripherally and two MT singlets or amorphous material with higher electron density (darker) at the center of axonemes. This is demonstrated in a transverse section of WT vestibular kinocilia (Figure 3C, indicated by an arrow). The “9+0” configuration lacks the central MTs structure and electron-dense materials as seen in the *Camsap3*-cKO image in Figure 3D. More than 71% of vestibular kinocilia from WT mice have “9+2” configuration, while only 24% vestibular kinocilia from *Camsap3*-cKO mice showed similar structure. The majority of *Camsap3*-cKO vestibular kinocilia (55%) have the “9+0” configuration. In addition, irregular configurations, “8+2” and “8+0,” were found in both WT (12%) and *Camsap3*-cKO (21%). Distribution of the MT configurations between WT and KO was statistically significant different as analyzed using a Kolmogorov-Smirnov distribution comparison test (*p* = 0.0003). Our data suggests that vestibular kinocilia of *Camsap3*-cKO were more likely missing the central MT pair than WT vestibular kinocilia.

Kinocilia are known to be essential for establishing the orientation of stereocilia bundles on hair cells. Data collected from P23 to 13-month showed that vestibular kinocilia were present on the vestibular hair cells of *Camsap3*-cKO mice just as their WT littermates. In addition, the shape and orientation of stereocilia on vestibular hair cells from *Camsap3*-cKO were quantitatively similar to those on WT mice despite the vestibular kinocilia from *Camsap3*-cKO mice were shorter and more likely to miss the central MT pair (Figures 2, 3). We also investigated the impact of CAMSAP3 on the orientation of stereocilia bundles by comparing auditory hair cells from WT and *Camsap3*-cKO mice with matched ages and sexes. As shown in Supplementary Figure 3, the orientation of stereocilia bundles on auditory hair cells from *Camsap3*-cKO mice were comparable to those on WT auditory hair cells. This data is not surprising because most of kinocilia on auditory hair cells are with “9+0” configuration (Sobkowicz et al., 1995), and CAMSAP3 signals do not co-localize with the basal bodies where “9+0” axonemes are originated (Robinson et al., 2020). In summary, CAMSAP3 is unlikely to have a significant impact on cochlear kinocilia and the orientation of stereocilia.

### Body Weight Differences Between Wildtype and *Camsap3*-cKO Mice Arise as Mice Age

**FIGURE 4:**
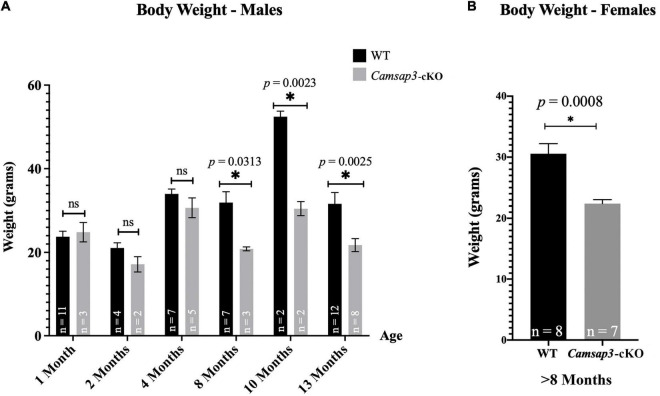
Weight differences between WT and *Camsap3*-cKO mice. **(A)** Male *Camsap3*-cKO mice weigh less than their WT counterparts after the age of 8-month, while no body weight difference was observed in mice younger than 4-month old. Mice were weighed prior to euthanasia at ages P36–P388. ns, not statistically significant difference. **(B)** Female *Camsap3*-cKO mice aged 8–13 months weigh less than their WT counterparts. For both male and female mice, the weights of old WT and *Camsap3*-cKO mice were significantly different. *p*-values for each age group of mice were as indicated. *Statistically significant difference.

In our original measurements of young mice, ranging from 1 to 4 months old, there was no weight difference between *Camsap3*-cKO mice and their WT littermates (Figure 4A). This phenotype differs from KD mice, *Camsap3-*KD, which were born smaller than their WT littermates (Robinson et al., 2020). Since we did not notice obvious body weight differences like those observed in the global *Camsap3-*KD mice, we assumed that their weights remained comparable. Though there were no obvious weight differences between WT and *Camsap3*-cKO mice when mice were less than 4-month old, weight differences emerged in 8-month old mice (Figure 4A). For mice 8 months and older (P237–P388), the average male WT mouse weighed (mean ± SD) 39.61 g ± 8.703 (*n* = 21), and the average male *Camsap3*-cKO mouse weighed 26.49 g ± 4.904 (*n* = 13). For female mice older than 8 months, the average WT mouse weighed 30.56 g ± 4.687 (*n* = 8), and the average female *Camsap3*-cKO mouse weighed 22.38 g ± 1.727 (*n* = 7). WT mice older than 8-month were significantly larger than the *Camsap3*-cKO mice regardless their sex (Figure 4).

### *Camsap3*-cKO Mice Do Not Have Notable Vestibular Dysfunction

Since CAMSAP3 was essential to build or maintain normal structure of vestibular kinocilia (Figures 2, 3), we further investigated whether shorter kinocilia on vestibular hair cells caused vestibular dysfunction. Vestibular dysfunction can manifest as issues with balance or gait abnormalities. In their home cages, *Camsap3*-cKO mice did not show obvious head tilt, walked normally, and were indistinguishable from their WT littermates. To assess the vestibular function of *Camsap3*-cKO mice, we performed multiple methods aiming to detect more subtle abnormalities. The simplest methods of testing vestibular functions include a swim test and a right-reflex test (Hardisty-Hughes et al., 2010). The swim test aims to determine if the mice could properly swim in a straight line and keep themselves upright. After assessing 26 WT (14 male and 12 female), and 27 *Camsap3*-cKO (14 male and 13 female) mice, ages ranging from P22 to P388, we found that there were no notable differences between the *Camsap3*-cKO and WT mice regarding their ability to swim. The right-reflex test assesses whether the mice can re-right themselves when flipped upside down. The same group of 26 WT and 27 *Camsap3*-cKO mice completed the right-reflex test. We found no difference in performance between the WT and *Camsap3-cKO* mice as each of the mice was able to flip themselves back to upright immediately (within 1 s). Subsequently, we analyzed the balance ability of *Camsap3*-cKO mice by rotarod apparatus. On the Rotarod, we assessed 12 WT (6 male and 6 female) and 8 *Camsap3*-cKO (5 male and 3 female) mice, ages P37–P153, and found that there was no significant difference in TTF between the two groups (data not shown).

### *Camsap3*-cKO Mice Have Slightly Altered Gaits

**FIGURE 5:**
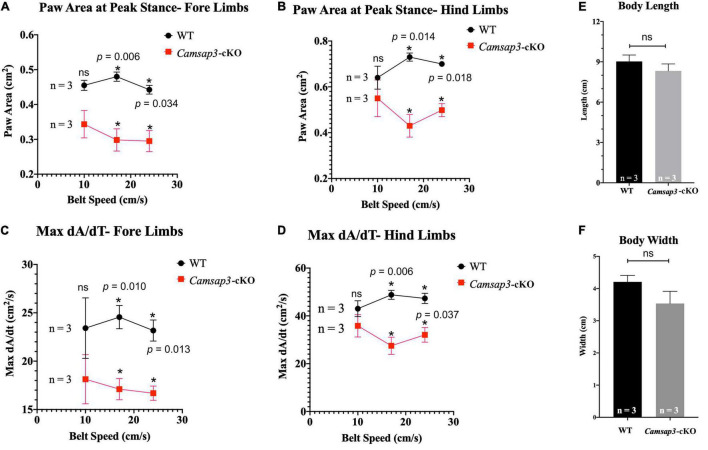
Gait differences between WT and *Camsap3*-cKO mice. Two litters of mice, ages P130–P133, were tested using the DigiGait Imaging System. Multiple *t*-tests revealed that Paw Area **(A,B)** and Max dA/dt **(C,D)** were significantly different between WT and *Camsap3*-cKO mice (*p*-values for each speed were as indicated). Bars represent mean ± SD. **(E)** Unpaired *t*-tests showed that there was no body length difference between male WT and *Camsap3*-cKO mice (ns, *p* = 0.1572). **(F)** Unpaired *t*-tests showed that there was also no body width difference between male WT and *Camsap3*-cKO mice (ns, *p* = 0.0539). *N* = 3 for both WT and *Camsap3*-cKO mice. *Statistically significant difference.

The vestibular function of *Camsap3*-cKO mice was also examined using the DigiGait Imaging System (Berryman et al., 2009). Since mice older than 8-month *Camsap3*-cKO mice were lighter than their WT littermates (Figure 4A), mice at 4 months old were selected for gait measurement. At this age, gait was fully developed (Akula et al., 2020; Rahn et al., 2021), and *Camsap3*-cKO mice were not significantly lighter than their WT littermates (Figure 4A). The assessed litter consisted of 6 male mice: 3 WT and 3 *Camsap3*-cKO. We collected data from these mice using the DigiGait™ Imaging System. Of all the aspects of gait analyzed by the DigiGait analysis software, most showed no significant difference between the WT and *Camsap3*-cKO mice. Some of the results were shown in the Supplementary Figures 1, 2). Two aspects of gait with significantly differences were paw area at peak stance and maximum dA/dt (Figure 5). Paw area (cm^2^) is the maximal paw area captured by the camera and corresponds with the time of “peak stance” (Supplementary Figure 1). When compared to their WT littermates, *Camsap3*-cKO mice had smaller areas of their paws in contact with the treadmill for both fore and hind limbs regardless of running speeds (Figures 5A,B). For example, at a speed of 17 cm/s, the paw area that *Camsap3*-cKO mice used to touch the treadmill was 62.1% (fore limbs) and 58.9% (hind limbs) of the paw area WT mice used. Maximum dA/dt (cm^2^/s) is the maximal rate of change of paw area in contact with the treadmill belt during the braking phase. At a low treadmill speed (10 cm/s), there was no substantial difference between the WT and *Camsap3*-cKO mice in their dA/dt for both fore and hind lambs. However, as the treadmill speeds increased, the difference between the WT and *Camsap3*-cKO mice became more apparent. At a high treadmill speed (24 cm/s), the maximum dA/dt decreased in *Camsap3*-cKO mice’s hind limbs (−10.5%), while WT mice had increased maximum dA/dt (+10.0%). Similar to paw area, maximum dA/dt in *Camsap3*-cKO mice were always slower than their WT littermates independent of walking speed. These differences exist despite there being no difference in the body length, width (Figures 5E,F), and weight (Figure 4A) between the WT and *Camsap3*-cKO mice.

### Older *Camsap3*-cKO Mice Have Renal Abnormalities

**FIGURE 6:**
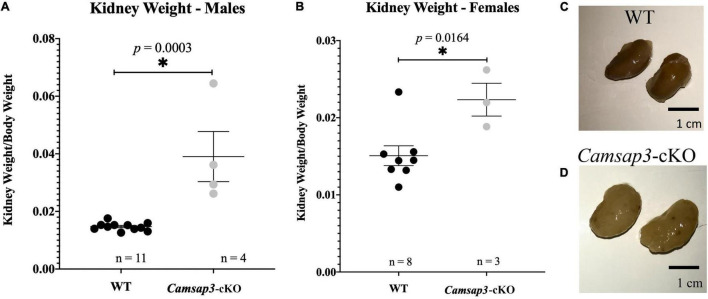
Renal abnormalities in *Camsap3*-cKO mice. **(A,B)** The kidney-to-body weight ratio in male **(A)** and female **(B)**. Unpaired *t*-tests revealed that *Camsap3*-cKO mice (P22-P339) showed a statistically significant increase in their kidney-to-body weight ratio relative to their WT counterparts. *p*-values for each age group of mice were as indicated. Bars represent mean ± SD. **(C,D)** Representative kidney images taken from male, 1-year-old WT **(C)** and *Camsap3*-cKO littermate **(D)**. Kidneys from the *Camsap3*-cKO mouse were hypertrophic and discolored. *Statistically significant difference.

In Tg*Pax2^Cre^* mouse line, *Cre* was detected in the embryonic kidney since the 12-somite stage to remove the floxed allele (Ohyama and Groves, 2004). Since CAMSAP3 is essential to orienting the apical-to-basal polarity of MT arrays in epithelial cells (Toya et al., 2016), we suspected that kidney function might be affected in *Camsap3*-cKO mice. Therefore, both kidneys were prepared out of euthanized WT and cKO mice with ages of P22–P339. Since the kidney-to-body weight ratio has been commonly used to predict kidney function (Hughes et al., 1999; Mitsuhata et al., 2021), we weighed 19 WT mice and 7 *Camsap3*-cKO mice and their kidneys. Kidney weight as a proportion of body weight was recorded. Female mice that had previously given birth were excluded. Interestingly, we found that *Camsap3*-cKO mice, especially the males, had statistically significant heavier kidneys (as a function of body weight) than their WT littermates (Figure 6). In male WT mice, their kidneys made up (mean ± SD) 1.47 ± 0.13% of their body weight, and in male *Camsap3*-cKO mice, their kidneys equaled 3.90 ± 1.5% of their body weight (Figure 6A). In female WT mice, their kidneys made up 1.51 ± 0.34% of their body weight, while in female *Camsap3*-cKO mice, their kidneys equaled 2.23 ± 0.30% of their body weight (Figure 6B). Kidneys from the *Camsap3*-cKO mice were hypertrophic and discolored (Figure 6C). Such a phenotype was similar to those observed in CAMSAP3-mutant knocking in mice model *Camsap3^dc/dc^*, in which exons 14–17 of *Camsap3* were removed from the genome of *Camsap3^dc/dc^* mice (Toya et al., 2016). Exons 14–17 encode the key MT binding domain of CAMSAP3, CKK domain. Because the mutant CAMSAP3 without the CKK domain is incapable of binding to MTs to form proper MT network in the epithelial cells in kidney, *Camsap3^dc/dc^* mice developed malfunction of kidneys with cyst at the proximal convoluted tubules starting at E17.5 even though kidney had normal appearance at P21 (Mitsuhata et al., 2021). Since renal dysfunction often caused body weight loss (Hickman and Swan, 2010), the deterioration of kidneys observed in *Camsap3*-cKO may contribute to the small body size of *Camsap3*-cKO mice older than 8-month (Figure 4). In consequence, health issues arose, and these mice became thin, hunched, and moved slowly. These health issues prevented further investigation of CAMSAP3’s impact on vestibular function in *Camsap3*-cKO mice that were older than 13 months of age.

### *Camsap3*-cKO Mice Maintain Normal Olfaction

**FIGURE 7:**
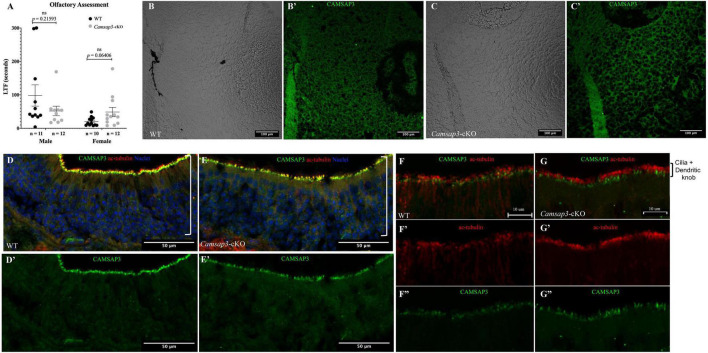
WT and *Camsap3*-cKO mice show no differences in olfaction. **(A)** No differences in the reaction time of the sense of smell between WT and *Camsap3*-cKO mice. Bars represent mean ± SD. Unpaired *t*-tests revealed no significant (ns) differences between WT and *Camsap3*-cKO mice in male or female mice. **(B,C)** CAMSAP3 had little expression in olfactory bulbs regardless of genotype, WT **(B)** or *Camsap3*-cKO **(C)**. The representative images were taken from female WT and their *Camsap3*-cKO littermates at P180. **(B’,C’)** were the cells shown in **(B,C)** stained with anti-Camsap3 (green). **(D–G)** CAMSAP3 was expressed in olfactory sensory neurons (OSN) in both WT and *Camsap3*-cKO mice. Olfactory mucosa in the nasal cavity from WT **(D,F)** and *Camsap3*-cKO **(E,G)** mice were stained with anti-Camsap3 (green), anti-acetylated tubulin (red, olfactory cilia, and nerve fibers), and Hoechst 33342 (blue, nuclei). **(F,G)** were images with higher magnification showing olfactory cilia and dendritic knobs layers on OSNs. **(D’,E’,F”,G”)** Showed the anti-CAMSAP3 (green) channel in **(D–G)**. **(F’,G’)** Showed anti-acetylated tubulin (red) channel in **(F,G)**. “[” Marks the OSN layer in the olfactory epithelium. Scale Bars = 100 μm **(B,C)**, 50 μm **(D,E)**, 10 μm **(F,G)**.

Since *Cre* was expressed in the olfactory bulb in Tg*Pax2^Cre^* mouse line to remove the floxed *Camsap3* exon 7, we investigated whether *Camsap3*-cKO had normal olfaction. Olfaction evaluation was performed for WT (11 male and 10 female) and *Camsap3*-cKO mice (12 male and 12 female) from 8 litters of mice, ages P55–P313. Mice were placed in a large container filled with bedding, and their latency to find (LTF) a buried Nutter Butter cookie was recorded. As shown in Figure 7A, WT and textitCamsap3-cKO mice took equal amounts of time to find the buried cookie. There was no significant difference between WT and *Camsap3*-cKO mice regardless their sex. We then examined CAMSAP3 expression in the olfactory bulb. Olfactory bulbs from P180 WT and *Camsap3*-cKO littermates were collected and stained with anti-Camsap3. As shown in Figures 7B,C, there was little CAMSAP3 expression in cells located in olfactory bulbs from both the WT and *Camsap3*-cKO mice, suggesting that CAMSAP3 has a minimal role in olfactory bulbs. We also compared CAMSAP3 expression in olfactory sensory neurons, where CAMSAP3 was abundantly expressed (Robinson et al., 2020). As shown in Figures 7D–G, CAMSAP3 was abundantly expressed in olfactory sensory neurons located in the nasal olfactory epithelia in both the WT and *Camsap3*-cKO mice. CAMSAP3 expression in olfactory sensory neurons is not affected in *Camsap3*-cKO mice and remained concentrated at dendritic knobs under the olfactory cilia (Figures 7F,G). In other words, CAMSAP3 expression in cells involved in olfaction were not changed in *Camsap3*-cKO mice compared to their WT littermates. Given the equivalent CAMSAP3 expression in cells involved in olfaction (Figures 7B–G), it was unsurprising that the *Camsap3*-cKO mice performed equally as well as WT mice on the open-field olfaction assessment.

## Discussion

Kinocilia on vestibular hair cells have MTs arranged in a “9+2” configuration that differs from most primary sensory cilia. Unlike the central MT pair in motile cilia, the function of the central MT pair in primary sensory cilia, including vestibular kinocilia, remains unknown. Our data shows that CAMSAP3 is located at the base of vestibular kinocilia (Figure 3). Removing or reducing CAMSAP3 in vestibular hair cells leads to abnormal kinocilia, which have shorter axonemes (Figure 2) and are more often missing the central MT pair (Figure 3). Since CAMSAP3 is required for *de novo* formation of the central MT pair for the “9+2” configuration in motile cilia (Robinson et al., 2020; Saito et al., 2021), it is likely that lacking CAMSAP3 and CAMSAP3-stabilized MTs in vestibular hair cells also prevents the central MT pair formation in vestibular kinocilia. This likely leads to the shortened vestibular kinocilia observed in *Camsap3*-cKO mice (Figure 2). In addition, *Camsap3*-cKO mice have more irregular configurations (8+2, 8+0) in vestibular kinocilia than their WT littermates. Although a very small portion of irregular MT configuration is commonly found in WT cilia, an increased proportion of irregular MT configurations in cilia was often associated with damage or degeneration of cilia (Piorunek et al., 2008). With increased irregular MT configurations and decreased “9+2” configuration in vestibular kinocilia from *Camsap3*-cKO, our data suggests that the central MT pair in vestibular kinocilia is important in establishing and/or maintaining the long axonemes observed in vestibular kinocilia.

Despite their abnormal vestibular kinocilia formation, *Camsap3*-cKO mice do not have notable vestibular dysfunction such as slower right-reflexes, inferior balance, and gait asymmetries (Hardisty-Hughes et al., 2010; Lu et al., 2011; Kopecky et al., 2012; Mathur et al., 2015). Such incoherent results between morphological data and behavioral assays are not uncommon, as many other mutant mice with damaged hair cells or neurons in the vestibular organ showed no detectable vestibular dysfunction (Jones and Jones, 2014). Since balance is achieved and maintained by multiple systems including the vestibular system, vision, proprioception, and central nervous system, it is possible that the *Camsap3*-cKO mice are able to retain normal vestibular function because of compensation or adaptation by the central nervous system and other peripheral systems. In addition, the central MT pairs are not completely eliminated from all kinocilia on vestibular hair cells from *Camsap3*-cKO mice as shown in Figure 3E. We suspect that *Camsap3* might not be completely removed from all cells in the vestibular organ. It is also possible that CAMSAP3’s role is compensated for by other members of CAMSAP family, such as CAMSAP2, which compensates for CAMSAP3 to maintain MT networks (Tanaka et al., 2012). Nevertheless, *Camsap3*-cKO mice did show slightly altered gaits in their paw area at peak stance and maximum dA/dt. Assessment of paw placement during walking is often used to evaluate pain (Griffioen et al., 2015; Deuis et al., 2017), pain-killer medicines (Brings et al., 2021), diseases (Bernardes and Oliveira, 2017), and recovery of injuries including both peripheral and central nerve injury (Neumann et al., 2009; Heinzel et al., 2020). Mice with numbers of disorders decrease their paw area to form “tip-toe walking” patterns (Furusawa et al., 1996; Moskovitz et al., 2001). Such phenotypes are also found in humans, particularly in children. An underdeveloped or poor vestibular system is believed to contribute to some of the idiopathic toe walking cases observed in children (Montgomery and Gauger, 1978; Chu and Anderson, 2020). *Camsap3*-cKO mice also decreased their paw area in both hind paws that come into contact with the surface (Figure 5). It is, therefore, tempting to speculate that altered gaits observed in *Camsap3*-cKO mice is the consequence of shorter vestibular kinocilia due to a lack of CAMSAP3 and central MTs in their axoneme. However, vascular calcification can also cause tiptoe walking in mice (Okawa et al., 1998), and kidney disease is one etiology for vascular calcification (Palit and Kendrick, 2014). Although there is no evidence suggesting that *Camsap3* expression is changed in the somatosensory system of *Camsap3*-cKO mice, but the midbrain and cerebellum may reduce CAMSAP3 expression due to *Cre* expression in these issues. Changed CAMSAP3 expression in the midbrain and cerebellum may interfere with the somatosensory information process that might lead to alternated gaits in *Camsap3*-cKO.

Balance disequilibrium is a significant contributor to falls in the elderly. The most common cause of balance dysfunction is due to abnormal hair cells from the vestibular sensory epithelia of the vestibular organ. Because of malfunction of kidneys in *Camsap3*-cKO mice, we were not able to investigate the impact of CAMSAP3 on the vestibular function in older mice due to their health issues. Different *Camsap3* transgenic mouse models without damaged kidneys may be needed to investigate impact of CAMSAP3 and the central MT pair of kinocilia on vestibular function. In addition, it might be worthwhile to examine the vestibulo-ocular reflex (VOR) of *Camsap3* transgenic mice using video-oculography. Video-oculography quantifies eye movement data to evaluate vestibular function, and could highlight vestibular issues that we were not able to capture in this study.

In summary, our data suggest that CAMSAP3 is critical for construction of the central MT pair in vestibular kinocilia, which may be essential for building long and stable axonemes in these kinocilia on vestibular hair cells. Whether abnormal vestibular kinocilia of *Camsap3*-cKO mice could directly lead to vestibular function requires further investigation.

## Data Availability Statement

The original contributions presented in this study are included in the article/Supplementary Material, further inquiries can be directed to the corresponding author.

## Ethics Statement

The animal study was reviewed and approved by the Institutional Animal Care and Use Committees of Northwestern University.

## Author Contributions

JZ designed and performed some of the experiments and analyzed the data. JO’D performed some of the experiments and analyzed some of the data. JO’D and JZ wrote the manuscript. Both authors contributed to the article and approved the submitted version.

## Conflict of Interest

The authors declare that the research was conducted in the absence of any commercial or financial relationships that could be construed as a potential conflict of interest.

## Publisher’s Note

All claims expressed in this article are solely those of the authors and do not necessarily represent those of their affiliated organizations, or those of the publisher, the editors and the reviewers. Any product that may be evaluated in this article, or claim that may be made by its manufacturer, is not guaranteed or endorsed by the publisher.
