# Supplementary material for: Vestibular Hair Cells Require CAMSAP3, a Microtubule Minus-End Regulator, for Formation of Normal Kinocilia
Source: Front Cell Neurosci. 2022 Jun 17;16:876805. doi: 10.3389/fncel.2022.876805 (PMC9247359; doi:10.3389/fncel.2022.876805)
Supplement: Supplementary file 2 [file Data_Sheet_1.pdf]

Supplemental Materials

Vestibular Hair Cells Require CAMSAP3, a Microtubule Minus-End Regulator, for Formation of Normal Kinocilia

Josephine O'Donnell and Jing Zheng

Movie S1. A video recording of a WT mouse on the DigiGait Apparatus, played at half speed. The mice were recorded from the ventral view.

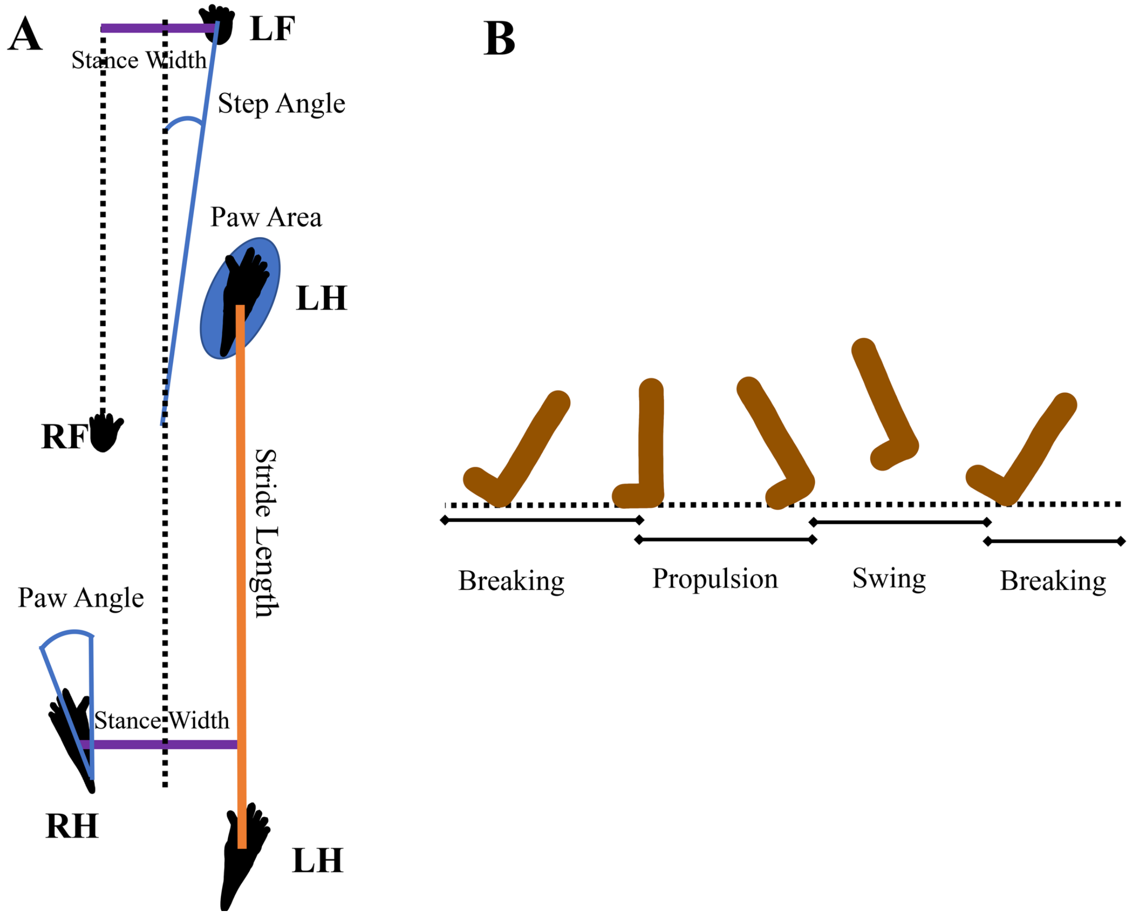

Supplemental Figure S1. A pictorial depiction of the DigiGait parameters. **A.** Ventral view of the four paws and key gait parameters: stance width, stride length, step angle, paw angle, and paw area. **B.** Side view of one paw throughout the course of one stride. Gait phases: breaking, propulsion, and swing. Stance width: The perpendicular distance between the centroids of either set of axial paws during peak stance. Paw area: The area seen by the camera, and reported at the time corresponding to peak stance (e.g., maximal paw area).

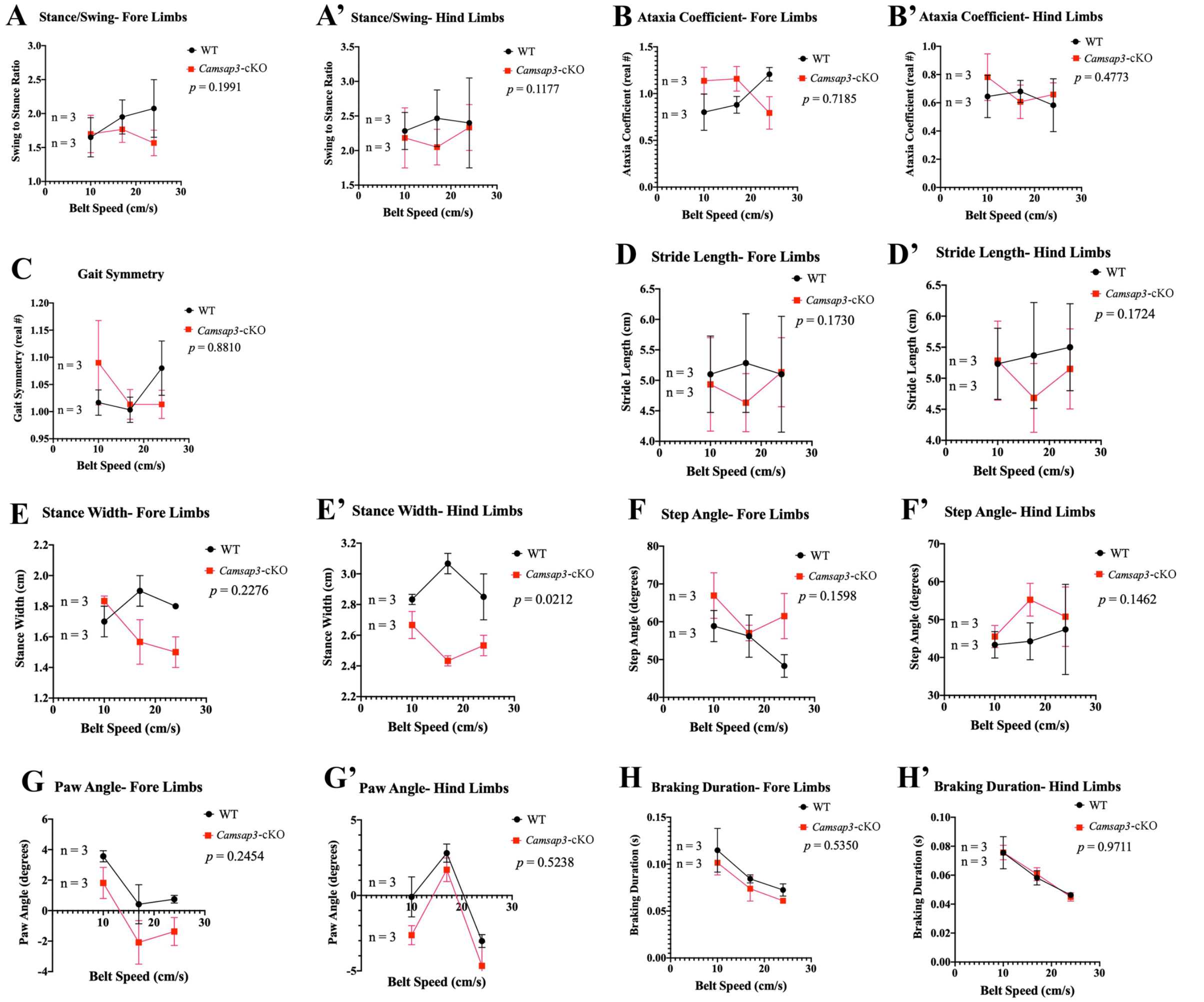

Supplemental Figure S2. Comparison of gaits. Two sets of littermates, P130-P133, were tested using the DigiGait^{TM} Imaging System. N = 3 for both WT and *Camsap3*-cKO mice. Bars represent mean ± SD. **A - A'**. Swing to stance ratio: the ratio of swing phase duration to stance phase duration for the fore limbs (A) and hind limbs (A'). Unpaired *t*-tests revealed that the swing to stance ratio was not significantly different between WT and *Camsap3*-cKO mice. p = 0.1991 (fore), p = 0.1177 (hind). **B - B'**. Ataxia coefficient: the measure of step-to step variability for the fore limbs (B) and hind limbs (B'). Unpaired *t*-tests revealed that the ataxia coefficient was not significantly different between WT and *Camsap3*-cKO mice. p = 0.7185 (fore), p = 0.4773 (hind). **C**. Gait Symmetry: the ratio of stepping frequency of fore limbs to hind limbs. An unpaired *t*-test revealed that the gait symmetry was not significantly different between WT and *Camsap3*-cKO mice. p = 0.8810. **D - D'**. Stride length: distance between steps of an individual paw for the fore limbs (D) and hind limbs (D'). Unpaired *t*-tests revealed that the stride length was not significantly different between WT and *Camsap3*-cKO mice. p = 0.1730 (fore), p = 0.1724 (hind). **E - E'**. Stance width: distance between fore (E) or hind limbs (E') laterally). Unpaired *t*-tests revealed that the stance width was not significantly different between WT and *Camsap3*-cKO mice for the fore limbs (p = 0.2276), but there was a significant difference in the hind limbs (p = 0.0212). **F - F'**. Step angle: the angle between the paws for the fore limbs (F) and hind limbs (F'). Unpaired *t*-tests revealed that the step angle was not

significantly different between WT and *Camsap3*-cKO mice. $p = 0.1598$ (fore), $p = 0.1462$ (hind). **G - G'**. Paw angle: the angle of the paw relative to the midline for the fore limbs (G) and hind limbs (G'). Unpaired *t*-tests revealed that the paw angle was not significantly different between WT and *Camsap3*-cKO mice. $p = 0.2454$ (fore), $p = 0.5238$ (hind). **H - H'**. Braking duration: duration of each stride in the braking phase for the fore limbs (H) and hind limbs (H'). Unpaired *t*-tests revealed that the breaking duration was not significantly different between WT and *Camsap3*-cKO mice. $p = 0.5350$ (fore), $p = 0.9711$ (hind).

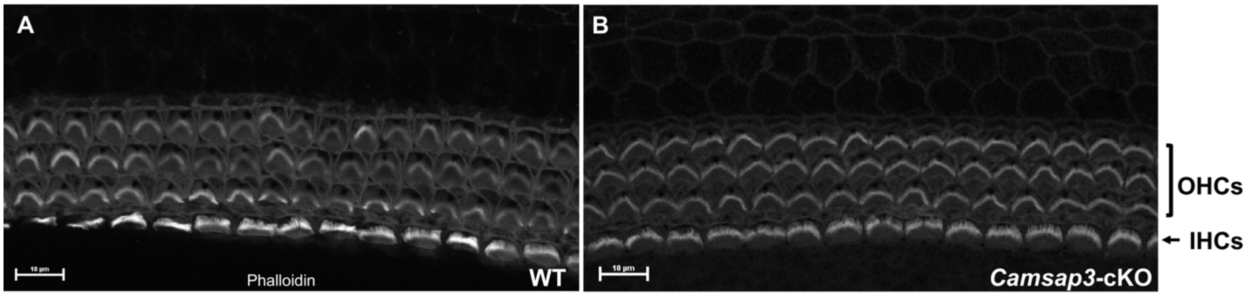

Supplemental Figure S3. The stereocilia orientation of auditory hair cells from of *Camsap3*-cKO mice appear normal. Representative immunofluorescent images of the organ of Corti from P4 WT (A) and *Camsap3*-cKO littermates (B) were shown. Scale Bars: 10 µm. Stereocilia on outer hair cells (OHCs) and inner hair cells (IHCs) from *Camsap3*-cKO were similar to those from WT.
